# MLF2 Negatively Regulates P53 and Promotes Colorectal Carcinogenesis

**DOI:** 10.1002/advs.202303336

**Published:** 2023-07-12

**Authors:** Debao Fang, Hao Hu, Kailiang Zhao, Aman Xu, Changjun Yu, Yong Zhu, Ning Yu, Bo Yao, Suyun Tang, Xianning Wu, Yide Mei

**Affiliations:** ^1^ Department of Thoracic Surgery, The First Affiliated Hospital of USTC Division of Life Sciences and Medicine University of Science and Technology of China Hefei Anhui 230001 China; ^2^ The CAS Key Laboratory of Innate Immunity and Chronic Disease Division of Life Sciences and Medicine University of Science and Technology of China Hefei Anhui 230027 China; ^3^ Department of General Surgery The First Affiliated Hospital of Anhui Medical University Hefei Anhui 230022 China; ^4^ Center for Advanced Interdisciplinary Science and Biomedicine of IHM Division of Life Sciences and Medicine University of Science and Technology of China Hefei Anhui 230027 China

**Keywords:** colorectal cancer, myeloid leukemia factor 2 (MLF2), p53, ubiquitin‐specific protease 7 (USP7)

## Abstract

Inactivation of the p53 pathway is linked to a variety of human cancers. As a critical component of the p53 pathway, ubiquitin‐specific protease 7 (USP7) acts as a deubiquitinase for both p53 and its ubiquitin E3 ligase mouse double minute 2 homolog. Here, myeloid leukemia factor 2 (MLF2) is reported as a new negative regulator of p53. MLF2 interacts with both p53 and USP7. Via these interactions, MLF2 inhibits the binding of USP7 to p53 and antagonizes USP7‐mediated deubiquitination of p53, thereby leading to p53 destabilization. Functionally, MLF2 plays an oncogenic role in colorectal cancer, at least partially, via the negative regulation of p53. Clinically, MLF2 is elevated in colorectal cancer and its high expression is associated with poor prognosis in patients with colorectal cancer. In wild‐type‐p53‐containing colorectal cancer, MLF2 and p53 expressions are inversely correlated. These findings establish MLF2 as an important suppressor of p53 function. The study also reveals a critical role for the MLF2–p53 axis in promoting colorectal carcinogenesis.

## Graphical abstract

Inactivation of the p53 tumor suppressive pathway is linked to a variety of human cancers. This study identifies myeloid leukemia factor 2 (MLF2) as a negative regulator of p53. MLF2 inhibits the binding of ubiquitin‐specific protease 7 (USP7) to p53 and antagonizes USP7‐mediated deubiquitination of p53, thereby leading to p53 destabilization. Functionally, MLF2 promotes colorectal carcinogenesis via the negative regulation of p53.

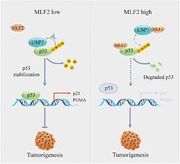

## 1 Introduction

p53 plays a prominent role in tumor prevention.^[^ ^1^, ^2^ ^]^ The tumor suppressive function of p53 is largely attributed to its ability to activate the expression of target genes that are involved in various cellular processes, such as cell cycle arrest, apoptosis, senescence, autophagy, ferroptosis, and cell metabolism.^[^ ^3^, ^4^, ^5^, ^6^, ^7^, ^8^ ^]^ In approximately half of human cancers, p53 is inactivated by either chromosomal deletion or somatic mutation. Even in tumors harboring wild‐type p53, the p53 pathway is often disrupted by altered expression of upstream or downstream regulatory factors.^[^ ^9^, ^10^ ^]^ Moreover, germline mutations of p53 have been linked to Li‐Fraumeni syndrome, a cancer predisposition disorder.^[^ ^11^ ^]^ Therefore, inhibition of p53 activity is considered to be crucial for tumorigenesis.

Given the potent antiproliferative activity of p53, its expression needs to be tightly restricted in unstressed cells. It has been well accepted that p53 expression is mainly regulated at the level of protein stability, which allows rapid p53 accumulation and activation upon stress. Under normal conditions, p53 is expressed at low levels due to the constant ubiquitination and proteasomal degradation mediated by several ubiquitin E3 ligases, among which mouse double minute 2 homolog (Mdm2) is the major ubiquitin E3 ligase for p53.^[^ ^12^, ^13^, ^14^, ^15^ ^]^ The physiological importance of Mdm2 in suppressing p53 expression is highlighted by the observation that the embryonic lethality of Mdm2‐null mice can be fully rescued by simultaneous p53 deletion.^[^ ^16^, ^17^ ^]^ Under stressed conditions, Mdm2‐mediated p53 degradation is compromised, thereby leading to p53 stabilization and activation.^[^ ^18^ ^]^

Ubiquitin‐specific protease 7 (USP7), also known as herpesvirus‐associated ubiquitin‐specific protease, is a critical regulator of the Mdm2–p53 pathway.^[^ ^19^, ^20^ ^]^ Both Mdm2 and p53 can bind to the tumor necrosis factor receptor‐associated factor (TRAF)‐like domain in the N‐terminus of USP7 in a mutually exclusive manner.^[^ ^21^ ^]^ USP7 is able to deubiquitinate both Mdm2 and p53 and has a dynamic role in controlling p53 expression.^[^ ^20^, ^22^ ^]^ Complete ablation of USP7 results in Mdm2 destabilization and subsequent p53 stabilization, possibly because USP7 binds to Mdm2 with a higher affinity than p53.^[^ ^21^, ^23^, ^24^, ^25^ ^]^ Paradoxically, however, overexpression of USP7 can also stabilize p53 even in the presence of Mdm2.^[^ ^26^ ^]^ These findings indicate the importance and complexity of USP7 in the regulation of the Mdm2–p53 signaling. Therefore, identification and functional characterization of new regulators of the deubiquitinating activity of USP7 toward Mdm2 or p53 would be of great importance to the detailed understanding of p53 biology.

Myeloid leukemia factor 2 (MLF2) is a member of the myeloid leukemia factor family.^[^ ^27^ ^]^ Compared to its homolog MLF1, MLF2 is less functionally characterized. MLF2 has been shown to interact with mutant huntingtin (HTT) protein and suppress HTT aggregation and toxicity.^[^ ^28^ ^]^ In addition, MLF2 is a luminal component of the nuclear envelope blebs and can decrease the accumulation of phenylalanine–glycine repeat‐containing nucleoporins.^[^ ^29^, ^30^ ^]^ Besides, MLF2 may act as an oncogenic factor in breast cancer and chronic myelogenous leukemia.^[^ ^31^, ^32^ ^]^ However, it remains unknown how MLF2 contributes to tumorigenesis.

In the present study, we show that MLF2 is a negative regulator of p53. MLF2 is able to destabilize p53 in a USP7‐dependent manner. Mechanistically, MLF2 binds to USP7 and p53 and disrupts the USP7–p53 interaction. This disassociation attenuates USP7‐mediated p53 deubiquitination and causes p53 destabilization. Functionally, MLF2 was shown to promote colorectal carcinogenesis via p53 inhibition. Moreover, MLF2 is overexpressed in clinical colorectal cancer specimens and high MLF2 expression is associated with reduced patient survival. Together, these findings support that MLF2 is an important suppressor of p53 function and implicate MLF2 as a potential therapeutic target for colorectal cancer.

## 2 Results

### 2.1 MLF2 Is a p53‐Interacting Protein

To better understand how p53 is regulated, we sought to identify novel p53‐interacting proteins. HCT116 cells were lysed and immunoprecipitated with anti‐p53 antibody or an isotype‐matched control immunoglobulin G (IgG). The immunoprecipitated proteins were then subjected to mass spectrometry analysis. MLF2 was identified in anti‐p53 immunoprecipitates (Table S1, Supporting Information).

**Figure 1:**
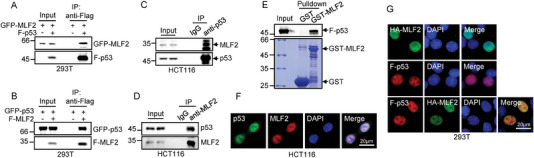
MLF2 interacts with p53. A) HEK293T cells were transfected with either GFP–MLF2 alone or together with Flag–p53. B) HEK293T cells were transfected with either GFP–p53 alone or together with Flag–MLF2. 24 h later, cell lysates were subjected to immunoprecipitation analysis. C,D) Lysates from HCT116 cells were immunoprecipitated with anti‐p53 antibody (C) or anti‐MLF2 antibody (D), and isotype‐matched IgG was used as a control. E) Recombinant GST or GST–MLF2 proteins immobilized on glutathione beads were incubated with purified Flag–p53. Input and bead‐bound proteins were analyzed by western blotting. F) Immunofluorescence staining of HCT116 cells with the indicated antibodies. The images were taken with a fluorescence microscope (Leica DMI600 B). Scale bar, 20 µm. G) Immunofluorescence staining of HEK293T cells transfected with HA–MLF2 (green), Flag–p53 (red), or both HA–MLF2 (green) and Flag–p53 (red). The images were taken with a fluorescence microscope (Leica DMI600 B). Scale bar, 20 µm.

To confirm the interaction between MLF2 and p53, we ectopically expressed MLF2 and p53 in human embryonic kidney 293T (HEK293T) cells. The subsequent reciprocal immunoprecipitation assay revealed a specific interaction of these two exogenously expressed proteins (**Figure** **1**A,B). This interaction of exogenously expressed MLF2 and p53 was also supported by a proximity ligation assay (PLA) (Figure S1A, Supporting Information). The endogenous interaction between MLF2 and p53 was further verified using both a co‐immunoprecipitation assay with anti‐p53 antibody and a reciprocal assay with anti‐MLF2 antibody (Figure 1C,D). Moreover, MLF2 appeared to directly interact with p53, as shown by an in vitro binding assay with purified glutathione‐S‐transferase (GST) tagged MLF2 and Flag–p53 proteins (Figure 1E). The immunofluorescence assay showed that both endogenous and exogenous MLF2 and p53 were colocalized in the nucleus (Figure 1F,G). Together, these data demonstrate that MLF2 is a novel binding partner for p53.

To identify the region of MLF2 that is responsible for the interaction with p53, we generated two MLF2 deletion mutants (Figure S1B, Supporting Information). The N‐terminal region (aa 1–150) of MLF2 strongly associated with p53, whereas the C‐terminal fragment (aa 151–248) of MLF2 exhibited no interaction with p53 (Figure S1C, Supporting Information). To delineate the MLF2‐binding domain in p53, we also generated a panel of p53 deletion mutants (Figure S1D, Supporting Information). Both p53 (∆aa 117–274) and p53 (aa 1–296) strongly bound to MLF2, whereas p53 (aa 61–393) showed no binding to MLF2 (Figure S1E, Supporting Information), suggesting that the transactivation domain of p53 likely mediates the interaction with MLF2.

### 2.2 MLF2 Suppresses p53 Expression by Enhancing Its Ubiquitin‐Dependent Degradation

**Figure 2:**
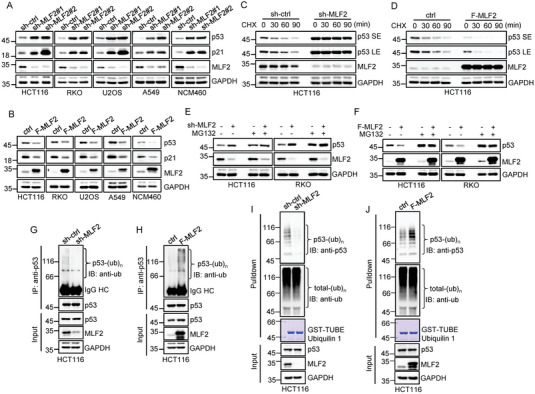
MLF2 suppresses p53 expression by enhancing its ubiquitin‐dependent degradation. A) The indicated cells were infected with lentiviruses expressing control shRNA, MLF2 shRNA#1, or MLF2 shRNA#2. B) The indicated cells were infected with lentiviruses expressing control or Flag–MLF2. 48 h later, cell lysates were analyzed by western blotting. C) HCT116 cells were infected with lentiviruses expressing control shRNA or MLF2 shRNA. D) HCT116 cells were infected with lentiviruses expressing control or Flag–MLF2. 48 h later, cells were treated with cycloheximide (CHX; 20 µg mL^−1^) for the indicated periods of time, followed by western blot analysis to measure the half‐life of p53. SE and LE indicate short‐time exposure and long‐time exposure, respectively. E) HCT116 cells were infected with lentiviruses expressing control shRNA or MLF2 shRNA. F) HCT116 cells were infected with lentiviruses expressing control or Flag–MLF2. 48 h later, cells were treated with or without MG132 (20 µm) for an additional 6 h, followed by western blot analysis. G) HCT116 cells were infected with lentiviruses expressing control shRNA or MLF2 shRNA. H) HCT116 cells were infected with lentiviruses expressing control or Flag–MLF2. 48 h later, cells were treated with MG132 (20 µm) for an additional 6 h, followed by an in vivo ubiquitination assay to examine p53 ubiquitination. I,J) HCT116 cells with knockdown (I) or overexpression (J) of MLF2 were treated with MG132 (20 µm) for 6 h. Cell lysates were then incubated with GST–TUBEs (ubiquilin 1) immobilized on glutathione beads. Input and bead‐bound proteins were analyzed by western blotting.

The interaction between MLF2 and p53 prompted us to investigate whether MLF2 could regulate p53 expression. Knockdown of MLF2 dramatically increased the protein levels of p53 and its target gene *p21* in p53 wild‐type cancer cell lines (HCT116, RKO, U2OS, and A549) and a normal human colon mucosal epithelial cell line (NCM460) (**Figure** **2**A). By contrast, ectopic expression of MLF2 in these cells markedly decreased p53 and p21 protein levels (Figure 2B). However, neither knockdown nor overexpression of MLF2 influenced the protein levels of p53 and p21 in SW480 cells harboring mutant p53 (Figure S2A,B, Supporting Information). The luciferase reporter assay showed that the transcriptional activity of p53 was negatively regulated by MLF2 (Figure S2C,D, Supporting Information). Moreover, knockdown of MLF2 elevated, whereas overexpression of MLF2 reduced, the messenger RNA (mRNA) levels of p53 target genes such as *p21*, TP53 induced glycolysis regulatory phosphatase (*TIGAR)*, p53 upregulated modulator of apoptosis (*PUMA)*, and *NOXA* (Figure S2E,F, Supporting Information). These data suggest that MLF2 is able to suppress p53 expression.

Given that MLF2 suppresses p53 expression without affecting its mRNA levels (Figure S2E,F, Supporting Information), we sought to evaluate whether MLF2 regulates the stability of p53 protein. MLF2 knockdown prolonged the half‐life of p53 (Figure 2C and Figure S2G (Supporting Information)), while MLF2 overexpression showed the opposite effect (Figure 2D and Figure S2H (Supporting Information)), implying that MLF2 accelerates the degradation of p53. In support of this notion, the inhibitory effect of MLF2 on p53 levels was diminished in the presence of the proteasome inhibitor MG132 (Figure 2E,F). Moreover, both immunoprecipitation and tandem‐repeated ubiquitin‐binding entities (TUBEs) pull‐down assays showed that knockdown of MLF2 prominently decreased, whereas overexpression of MLF2 significantly increased, p53 ubiquitination (Figure 2G–J and Figure S2I,J (Supporting Information)). Taken together, these data indicate that MLF2 destabilizes p53 by enhancing its ubiquitination and proteasomal degradation.

### 2.3 MLF2 Interacts with USP7 but Not Mdm2

**Figure 3:**
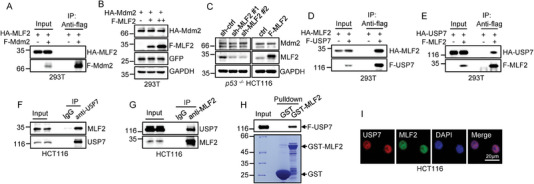
MLF2 interacts with USP7. A) HEK293T cells were transfected with either HA–MLF2 alone or together with Flag–Mdm2. 24 h later, cell lysates were subjected to immunoprecipitation analysis. B) HEK293T cells were transfected with either HA–Mdm2 alone or together with increasing amounts of Flag–MLF2. 24 h later, cell lysates were analyzed by western blotting. Levels of GFP and GAPDH were used as controls for transfection efficiency and sample loading, respectively. C) p53‐deficient (p53^−/−^) HCT116 cells were infected with the indicated lentiviruses. 48 h later, cell lysates were analyzed by western blotting. D) HEK293T cells were transfected with either HA–MLF2 alone or together with Flag–USP7. E) HEK293T cells were transfected with either HA–USP7 alone or together with Flag–MLF2. 24 h later, cell lysates were subjected to immunoprecipitation analysis. F,G) Lysates from HCT116 cells were immunoprecipitated with anti‐USP7 antibody (F) or anti‐MLF2 antibody (G), and isotype‐matched control IgG was used as a control. H) Recombinant GST or GST–MLF2 proteins immobilized on glutathione beads were incubated with purified Flag–USP7. Input and bead‐bound proteins were analyzed by western blotting. I) Immunofluorescence staining of HCT116 cells with the indicated antibodies. The images were taken with a fluorescence microscope (Leica DMI600 B). Scale bar, 20 µm.

We next investigated how p53 is destabilized by MLF2. Mdm2 is the primary ubiquitin E3 ligase for p53, which prompted us to test whether MLF2 could interact with Mdm2. The immunoprecipitation assay showed no interaction between MLF2 and Mdm2 (**Figure** **3**A). In addition, neither overexpression nor knockdown of MLF2 had an obvious impact on Mdm2 levels (Figure 3B,C). These findings indicate that the destabilizing effect of MLF2 on p53 is unlikely to be dependent on Mdm2.

It has been well recognized that the deubiquitinating enzyme USP7 is an important regulator of p53 stability. We therefore sought to determine whether USP7 is involved in the destabilizing effect of MLF2 on p53. We first examined the interaction of MLF2 and USP7 under overexpression conditions. MLF2 and USP7 were shown to interact with each other in both immunoprecipitation and PLA experiments (Figure 3D,E and Figure S3A (Supporting Information)). In addition, reciprocal co‐immunoprecipitation assays using either anti‐USP7 antibody or anti‐MLF2 antibody also verified the interaction between endogenous MLF2 and USP7 (Figure 3F,G). The GST pull‐down assay using purified GST–MLF2 and USP7 revealed that MLF2 directly associated with USP7 (Figure 3H). Furthermore, the immunofluorescence assay showed the colocalization of MLF2 and USP7 in the nucleus (Figure 3I and Figure S3B (Supporting Information)). Collectively, the above data suggest that MLF2 interacts with USP7 but not Mdm2.

To delineate the regions that are responsible for the interaction between MLF2 and USP7, we generated a series of deletion mutants of both MLF2 and USP7 and performed immunoprecipitation assays. MLF2 (aa 1–150) displayed a clear interaction, whereas MLF2 (aa 151–248) showed no interaction, with USP7 (Figure S3C, Supporting Information), suggesting that the N‐terminal region of MLF2 mediates the interaction with USP7. In addition, both USP7 (aa 506–1102) and USP7 (aa 1–208) associated with MLF2, although USP7 (aa 1–208) showed a lesser binding ability to MLF2 (Figure S3D, Supporting Information). Conversely, USP7 (aa 208–506) completely lost the binding capacity to MLF2 (Figure S3D, Supporting Information). Moreover, the ubiquitin‐like domains (UBL) 4, 5, but not UBL 1–3, of USP7 showed a strong interaction with MLF2 (Figure S3E, Supporting Information). These data imply that MLF2 likely binds to both N‐terminal TRAF‐like domain and the last two UBL 4, 5 in the C‐terminal region of USP7.

### 2.4 MLF2 Suppresses p53 Expression in a USP7‐Dependent Manner

**Figure 4:**
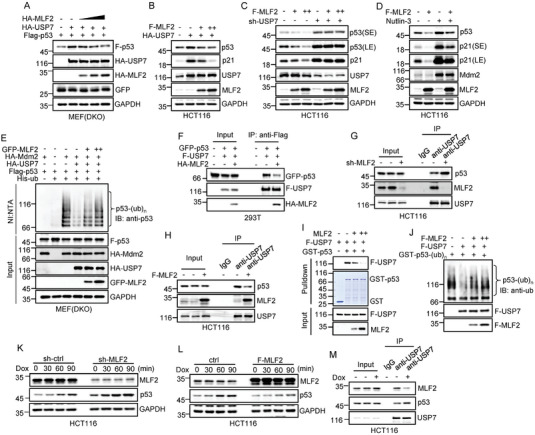
MLF2 suppresses p53 expression in a USP7‐dependent manner. A) Mdm2^−/−^p53^−/−^ MEF (double knockout, DKO) cells were transfected with Flag–p53, HA–USP7, and increasing amounts of HA–MLF2 as indicated. 24 h after transfection, cell lysates were subjected to western blot analysis. B) HCT116 cells were transfected with HA–USP7 alone or together with increasing amounts of Flag–MLF2. 24 h after transfection, cell lysates were subjected to western blot analysis. C) The control or USP7 knockdown HCT116 cells were infected with lentiviruses expressing Flag–MLF2 as indicated. 48 h later, cell lysates were subjected to western blot analysis. D) HCT116 cells were infected with lentiviruses expressing control or Flag–MLF2. 48 h later, cells were treated with or without 10 µm Nutlin‐3 for another 24 h, followed by western blot analysis. E) Lysates of Mdm2^−/−^p53^−/−^ MEF (DKO) cells transfected with plasmids encoding the indicated proteins were subjected to an in vivo ubiquitination assay. F) HEK293T cells were transfected with GFP–p53, Flag–USP7, and HA–MLF2 in the indicated combinations. 24 h later, cells were treated with MG132 (20 µm) for an additional 6 h, followed by immunoprecipitation analysis. G) HCT116 cells were infected with lentiviruses expressing either control shRNA or MLF2 shRNA. 48 h later, cells were treated with MG132 (20 µm) for an additional 6 h, followed by immunoprecipitation analysis. H) HCT116 cells were infected with lentiviruses expressing control or Flag–MLF2. 48 h later, cells were treated with MG132 (20 µm) for an additional 6 h, followed by immunoprecipitation analysis. I) Recombinant GST or GST–p53 proteins immobilized on glutathione beads were incubated with purified Flag–USP7 and increasing amounts of MLF2 as indicated. Input and bead‐bound proteins were analyzed by western blotting. J) Ubiquitinated GST–p53 purified from HEK293T cells was incubated with purified Flag–USP7 and increasing amounts of Flag–MLF2 as indicated. The reaction mixtures were analyzed by western blotting with antiubiquitin antibody. K,L) HCT116 cells with knockdown (K) or overexpression (L) of MLF2 were treated with doxorubicin (Dox; 0.5 µg mL^−1^) for the indicated periods of time. Cell lysates were then analyzed by western blotting. M) HCT116 cells were treated with MG132 (20 µm) for 5 h and with doxorubicin (Dox; 0.5 µg mL^−1^) or left untreated for an additional 1 h, followed by immunoprecipitation analysis.

To further determine whether MLF2 suppresses p53 expression via USP7, we ectopically expressed p53 together with USP7 or both USP7 and MLF2 in Mdm2^−/−^p53^−/−^ mouse embryonic fibroblast (MEF) cells. When USP7 was coexpressed with p53 in these cells, USP7 increased the expression levels of p53 as expected (**Figure** **4**A). However, this USP7‐increased expression of p53 was dose‐dependently reversed by MLF2 (Figure 4A). Similarly, the enhancing effect of USP7 on the expression levels of endogenous p53 and its target gene *p21* was diminished when MLF2 was concurrently overexpressed in HCT116 cells (Figure 4B). Moreover, MLF2 was shown to decrease p53 levels in control HCT116 cells but not in USP7 knockdown HCT116 cells (Figure 4C). These data indicate that MLF2 antagonizes the enhancing effect of USP7 on p53 expression. To rule out the involvement of Mdm2 in the inhibitory effect of MLF2 on p53 levels, we utilized the Mdm2‐specific inhibitor Nutlin‐3 to treat MLF2‐overexpressing HCT116 cells. Due to the existence of the autoregulatory feedback loop between p53 and Mdm2,^[^ ^33^, ^34^ ^]^ protein levels of both p53 and Mdm2 were increased upon Nutlin‐3 treatment (Figure 4D). It was evident that even in the presence of Nutlin‐3, the levels of p53 were still reduced by ectopic expression of MLF2 (Figure 4D), implying that it is unlikely that MLF2 inhibits p53 expression via Mdm2. Meanwhile, consistent with the above findings that MLF2 was able to eliminate the enhancing effect of USP7 on p53 expression (Figure 4A,B), USP7‐reduced p53 ubiquitination was greatly recovered by MLF2 (Figure 4E). Collectively, these findings suggest that MLF2 suppresses p53 expression by antagonizing USP7‐mediated deubiquitination of p53.

We next asked how MLF2 suppresses p53 expression via USP7. By performing an immunoprecipitation assay, we showed that the interaction between exogenously expressed USP7 and p53 was strongly reduced when MLF2 was simultaneously expressed (Figure 4F). In addition, MLF2 knockdown increased, whereas MLF2 overexpression decreased, the interaction between endogenous USP7 and p53 (Figure 4G,H), indicating that MLF2 inhibits the USP7–p53 binding. In support of this, MLF2 was shown to dose‐dependently decrease the association of USP7 and p53 in vitro (Figure 4I). Correlated with the inhibitory effect of MLF2 on the USP7─p53 binding, MLF2 was shown to compromise USP7‐catalyzed deubiquitination of p53 in vitro (Figure 4J). Together, these data suggest that MLF2 reduces the binding of USP7 to p53 and thus attenuates USP7‐mediated deubiquitination of p53, resulting in the destabilization of p53.

To further determine whether MLF2 regulates the p53 response to DNA damage, HCT116 cells with knockdown or overexpression of MLF2 were treated with the DNA‐damage‐inducing agent doxorubicin. Knockdown of MLF2 resulted in an earlier and stronger accumulation of p53 protein upon doxorubicin treatment (Figure 4K). By contrast, overexpression of MLF2 delayed the p53 response to doxorubicin treatment (Figure 4L). These data indicate that MLF2 indeed regulates DNA‐damage‐induced p53 response. By performing an immunoprecipitation assay, we also showed that when cells were treated with doxorubicin, the interaction between USP7 and MLF2 was significantly decreased, whereas the USP7–p53 interaction was noticeably increased (Figure 4M). Taken together, these findings imply that the decreased binding of MLF2 to USP7 may contribute to the p53 response to DNA damage.

### 2.5 MLF2 Negatively Regulates the Tumor Suppressive Activity of p53

**Figure 5:**
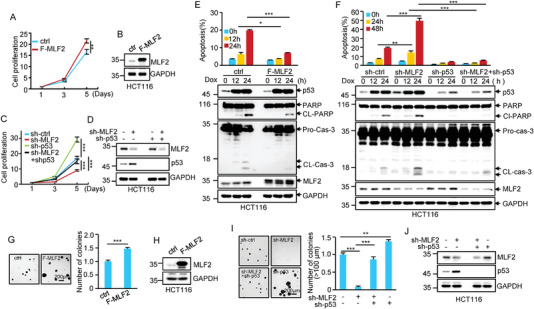
MLF2 negatively regulates p53 tumor suppressive activity. A) The growth curves of HCT116 cells expressing control or Flag–MLF2. B) The expression of MLF2 was detected by western blot analysis. Data shown are mean ± SD (standard deviation) (*n* = 3). **, *p* < 0.01. C) The growth curves of HCT116 cells expressing control shRNA, MLF2 shRNA, p53 shRNA, or MLF2 shRNA plus p53 shRNA. D) The knockdown efficiency of MLF2 and p53 was verified by western blot analysis. Data shown are mean ± SD (*n* = 3). ***, *p* < 0.001. E) HCT116 cells expressing control or Flag–MLF2 were treated with doxorubicin (Dox; 0.5 µg mL^−1^) for the indicated periods of time. F) HCT116 cells expressing control shRNA, MLF2 shRNA, p53 shRNA, or MLF2 shRNA plus p53 shRNA were treated with doxorubicin (Dox; 0.5 µg mL^−1^) for the indicated periods of time. Cells were costained with Annexin V–FITC and Hoechst 33342, and Annexin V‐positive cells were counted as apoptotic cells. Data shown are mean ± SD (*n* = 3). *, *p* < 0.05; ***, *p* < 0.001. Cell lysates were analyzed by western blotting with the indicated antibodies. CL‐PARP and CL‐Cas‐3 indicate cleaved poly(ADP‐ribosyl) polymerase (PARP) and cleaved caspase‐3, respectively. The representative images were shown in Figure S4E,H (Supporting Information). G) HCT116 cells expressing control or Flag–MLF2 were assayed for their ability to form colonies in soft agar. Images were taken 2 weeks after cell seeding. Numbers of colonies in six randomly selected areas (40 × magnification) were counted and averaged. H) The expression of MLF2 was also examined by western blot analysis. Data shown are mean ± SD (*n* = 3). ***, *p* ˂ 0.001. I) HCT116 cells expressing control shRNA, MLF2 shRNA, p53 shRNA, or MLF2 shRNA plus p53 shRNA were subjected to soft agar assay. Images were taken 2 weeks after cell seeding. Numbers of colonies in six randomly selected areas (40 × magnification) were counted and averaged. J) The knockdown efficiency of MLF2 and p53 was also verified by western blot analysis. Data shown are mean ± SD (*n* = 3). **, *p* ˂ 0.01; ***, *p* ˂ 0.001.

Given the inhibitory effect of MLF2 on p53 expression, we asked whether MLF2 could regulate the tumor suppressive activity of p53. We first evaluated the effect of MLF2 on cell proliferation and apoptosis in the colorectal cancer cell lines HCT116 and RKO. The results showed that overexpression of MLF2 in these cells resulted in accelerated cell proliferation (**Figure** **5**A,B and Figure S4A,B (Supporting Information)). Knockdown of MLF2 reduced the proliferation of HCT116 and RKO cells; however, this effect could be substantially reversed by the concurrent knockdown of p53 (Figure 5C,D and Figure S4C,D (Supporting Information)). In addition, MLF2 overexpression strongly decreased, whereas MLF2 knockdown dramatically increased, the sensitivity of HCT116 and RKO cells to doxorubicin‐induced apoptosis (Figure 5E,F and Figure S4E–J (Supporting Information)). This stimulatory effect of MLF2 knockdown on doxorubicin‐induced apoptosis was almost completely attenuated by the simultaneous knockdown of p53 (Figure 5F and Figure S4H–J (Supporting Information)). These data indicate that MLF2 regulates cell proliferation and apoptosis, at least partially, via the inhibition of p53.

We next examined the effect of MLF2 on the anchorage‐independent growth of HCT116 cells by performing a soft agar colony formation assay. Ectopic expression of MLF2 markedly increased, whereas knockdown of MLF2 strongly decreased, the number of colonies (Figure 5G–J). In addition, the reduced anchorage‐independent growth of HCT116 cells caused by MLF2 knockdown was markedly restored by p53 knockdown (Figure 5I,J). Collectively, these data suggest that MLF2 is able to negatively regulate the tumor suppressive activity of p53.

To further evaluate whether the promoting effect of MLF2 on cell proliferation is fully dependent on p53, SW480 cells harboring mutant p53 were used. The results showed that ectopic expression of MLF2 promoted, whereas knockdown of MLF2 inhibited, the proliferation and anchorage‐independent growth of SW480 cells (Figure S4K–P, Supporting Information), indicating that MLF2 regulates cell proliferation via both p53‐dependent and ‐independent mechanisms.

### 2.6 Biological Implication of the MLF2–p53 Axis in Colorectal Carcinogenesis

**Figure 6:**
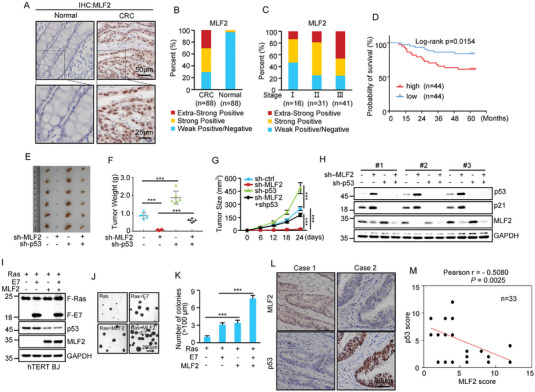
MLF2 facilitates colorectal carcinogenesis via the inhibition of p53. A) Representative immunohistochemistry images of MLF2 expression in colorectal adenocarcinoma (CRC) (*n* = 88) and matched adjacent normal tissues (*n* = 88). B) Quantification of MLF2 protein levels in CRC and matched adjacent normal tissues. The MLF2 expression levels were classified into four grades: negative, score 0–3; weak positive, score 4–6; strong positive, score 7–9; and extrastrong positive, score 10–12. C) Quantification of MLF2 protein levels in CRC from patients with different clinical stages. D) Kaplan–Meier curve showing the overall survival of CRC patients with high or low MLF2 expression. E–H) A total of 2 × 10^6^ HCT116 cells transduced with lentiviruses expressing control shRNA, MLF2 shRNA, p53 shRNA, or MLF2 shRNA plus p53 shRNA were individually injected into nude mice (*n* = 6 for each group). Xenograft tumors were taken 24 days after injection (E). Excised tumors were weighed (F). Tumor sizes were measured at the indicated time points (G). Protein extracts from the excised xenografts were also analyzed by western blotting (H). ***, *p* < 0.001. I–K) hTERT‐immortalized BJ cells were sequentially infected with lentiviruses expressing MLF2, E7, and Kras G12V in the indicated combination. 48 h later, cell lysates were analyzed by western blotting (I). These cells were also subjected to soft agar colony formation assay. Images were taken 3 weeks after cell seeding (J). Numbers of colonies in six randomly selected areas (40 × magnification) were counted and averaged (K). Data shown are mean ± SD (*n* = 3). ***, *p* ˂ 0.001. L,M) Representative immunohistochemistry images of MLF2 and p53 expression in 33 human colorectal adenocarcinoma samples harboring wild‐type p53 (L), and the Pearson correlation of staining intensity between MLF2 and p53 (M). Note that the scores of some samples overlapped.

The negative regulation of p53 tumor suppressive activity by MLF2 prompted us to ask whether MLF2 could function as an oncogenic molecule. By performing immunohistochemistry analysis of human colorectal adenocarcinoma and matched adjacent normal tissues, we showed that nearly 70% of colorectal adenocarcinoma samples exhibited strong or extra‐strong positive staining for MLF2, whereas less than 5% of the adjacent normal colorectal tissues displayed strong positive MLF2 staining (**Figure** **6**A,B). In addition, colorectal adenocarcinoma patients with a higher expression of MLF2 had a higher clinical stage (Figure 6C), indicating that the expression levels of MLF2 are positively correlated with the clinical stage of colorectal adenocarcinoma. Moreover, colorectal adenocarcinoma patients with high MLF2 expression had a shorter overall survival than those with low MLF2 expression (Figure 6D). These data indicate that MLF2 is a potential prognostic marker in colorectal cancer.

To investigate whether MLF2 exerts its oncogenic function via p53 inhibition, a xenograft mouse model was used. The results showed that MLF2 overexpression evidently promoted in vivo xenograft tumor growth of HCT116 cells, while MLF2 knockdown in HCT116 cells remarkably suppressed in vivo xenograft tumor growth (Figure 6E–H and Figure S5A–D (Supporting Information)). In addition, MLF2‐knockdown‐inhibited in vivo xenograft tumor growth was greatly recovered when p53 was concurrently knocked down (Figure 6E–H), suggesting that MLF2 facilitates in vivo colorectal cancer cell growth at least partially via the inhibition of p53.

Since MLF2 can negatively regulate p53 expression and inactivation of p53 is a crucial step for the in vitro transformation of human cells, we sought to utilize human‐telomerase‐reverse‐transcriptase (hTERT)‐immortalized human foreskin fibroblasts (BJ cells) to assess whether MLF2 could confer transformation potential to human cells. Lentiviruses expressing MLF2, the E7 protein of human papilloma virus type 16, which inactivates Rb, and the Kras^G12V^ mutant were sequentially introduced into hTERT‐immortalized BJ cells, followed by a soft agar colony formation assay. Compared to cells expressing Kras^G12V^ plus either E7 or MLF2, cells expressing all three proteins formed more and larger colonies in soft agar, accompanied by reduced levels of p53 (Figure 6I–K), suggesting that MLF2 promotes in vitro oncogenic transformation of human cells by inhibiting p53 expression.

To further validate the biological significance of MLF2‐suppressed p53 expression in colorectal cancer, we analyzed the expression levels of MLF2 and p53 in human colorectal adenocarcinoma harboring the wild‐type tumor protein p53 (*TP53)* gene. Intriguingly, in these wild‐type‐*TP53*‐containing colorectal adenocarcinoma samples, MLF2 protein levels were inversely correlated with p53 protein levels (Figure 6L,M). Taken together, these results strongly support that MLF2 promotes colorectal carcinogenesis via the negative regulation of p53.

## 3 Discussion

p53 is one of the most important tumor suppressor genes, and its inactivation has been implicated in more than half of human cancers.^[^ ^35^, ^36^ ^]^ Therefore, identification and functional characterization of new negative regulators of p53 is of great importance to the mechanistic understanding of tumorigenesis. In the current study, we present evidence showing that MLF2 destabilizes p53 by attenuating USP7‐mediated deubiquitination of p53. MLF2 is functionally shown to promote colorectal carcinogenesis via the negative regulation of p53. Thus, MLF2 is an important player in the regulation of p53 function.

It has been well recognized that the activity of p53 is mainly controlled by the ubiquitin E3 ligase Mdm2. Emerging evidence suggests that USP7 plays a dualistic and complicated role in regulating the Mdm2–p53 pathway,^[^ ^20^ ^]^ although the underlying mechanism is incompletely understood. On the one hand, USP7 can deubiquitinate and stabilize Mdm2, thereafter leading to p53 destabilization.^[^ ^23^, ^24^ ^]^ On the other hand, USP7 can also deubiquitinate and stabilize p53.^[^ ^22^, ^26^ ^]^ Here, we show that under nonstressed conditions, MLF2 destabilizes p53 in a Mdm2‐independent manner, as manifested by the findings that MLF2 is still capable of suppressing p53 expression even in the presence of the Mdm2 specific inhibitor Nutlin‐3. Mechanistically, by binding to UPS7, MLF2 disrupts the USP7–p53 interaction and reduces the deubiquitinating activity of USP7 toward p53, thereby resulting in the destabilization of p53. Intriguingly, the MLF2–USP7 interaction appears to be regulated upon DNA damage, as treatment of cells with doxorubicin greatly reduces the binding of MLF2 to USP7. We also show that knockdown of MLF2 promotes, whereas overexpression of MLF2 inhibits, the p53 response to DNA damage. These combined data imply that the dissociation of MLF2 from USP7 may contribute to DNA‐damage‐induced p53 response.

USP7 consists of seven domains, including an N‐terminal TRAF‐like domain, a central catalytic domain, and five C‐terminal UBL domains. A number of proteins have been reported to interact with USP7 and regulate its activity toward Mdm2 or p53.^[^ ^37^ ^]^ For instance, death domain associated protein (Daxx) promotes the binding of USP7 to Mdm2 and enhances Mdm2‐dependent p53 degradation.^[^ ^38^ ^]^ In addition, testis‐specific protein Y‐encoded like 5 (TSPYL5) and Epstein‐Barr virus nuclear antigen 1 (EBNA1) compete with p53 for binding to the TRAF‐like domain of USP7, thereby suppressing the function of p53.^[^ ^39^, ^40^ ^]^ Moreover, Abraxas brother 1 (ABRO1) stabilizes p53 by facilitating the interaction of p53 with USP7.^[^ ^41^ ^]^ In this study, we show that MLF2 binds to both N‐terminal TRAF‐like domain and C‐terminal UBL4‐5 of USP7. Since these two USP7 regions are also important for p53 binding,^[^ ^21^, ^25^, ^42^ ^]^ it is reasonable that MLF2 may disrupt the interaction between USP7 and p53 in a competitive manner.

MLF2 belongs to the myeloid leukemia factor family, which is involved in myelodysplastic syndrome and acute myeloid leukemia.^[^ ^43^, ^44^ ^]^ MLF2 and its homolog MLF1 share nearly 40% identity and are highly conserved in metazoans and mammals.^[^ ^44^, ^45^ ^]^ Unlike the destabilizing effect of MLF2 on p53, it has been previously reported that MLF1 stabilizes p53 by suppressing constitutive photomorphogenic 1 (COP1) via COP9 signalosome subunit 3.^[^ ^46^ ^]^ Therefore, it would be interesting to investigate the mechanisms underlying the opposite effects of MLF1 and MLF2 on p53 expression in the future. Although recent evidence suggests a potential oncogenic role of MLF2 in breast cancer and chronic myelogenous leukemia,^[^ ^31^, ^32^ ^]^ it remains unclear how MLF2 is involved in tumorigenesis.

In our study, we show that MLF2 acts as a suppressor of p53 function. Meanwhile, MLF2 is highly expressed in colorectal cancer and the expression of MLF2 is negatively correlated with patient survival. By using both in vitro cellular transformation assay and in vivo xenograft mouse model, MLF2 was shown to promote colorectal carcinogenesis via the inhibition of p53. Wild‐type‐p53‐containing colorectal cancer samples also exhibited an inverse correlation between MLF2 and p53 expression, indicating the physiological importance of MLF2‐mediated p53 suppression in colorectal cancer. These findings emphasize a critical role of the MLF2–p53 axis in the carcinogenesis of colorectal cancer with wild‐type p53. However, we should mention that MLF2 might also promote colorectal carcinogenesis via p53‐independent mechanism(s), since MLF2 is able to accelerate the proliferation of mutant‐p53‐bearing SW480 cells. Given that several p53‐activating compounds are currently under clinical trials for the treatment of cancers carrying wild‐type p53,^[^ ^47^, ^48^ ^]^ our findings of the inhibitory effect of MLF2 on p53 function therefore suggest MLF2 as a potential therapeutic target for cancer.

## 4 Experimental Section

### Reagents and Antibodies

The reagents and antibodies used in this study were purchased from the indicated sources: lipofectamine 2000 (Invitrogen), MG132 (APExBIO, 20 µm), complete ethylenediaminetetraacetic acid (EDTA)‐free protease inhibitor cocktail (APExBIO), anti‐Flag M2 affinity agarose gel (Sigma), protein A/G agarose beads (Thermo Fisher Scientific), cycloheximide (Sigma, 20 µg mL^−1^), polybrene (Sigma, 10 µg mL^−1^), doxorubicin (Sigma, 0.5 µg mL^−1^), 4′,6‐diamidino‐2‐phenylindole (DAPI) (Sigma, 1 µg mL^−1^), Hoechst 33342 (Sigma, 1 µg mL^−1^), 3 × Flag peptide (Sigma), *N*‐ethylmaleimide (Sigma), Annexin V–fluorescein isothiocyanate (FITC) (Sigma), glutathione beads (GE Healthcare), Nutlin‐3 (Sigma, 10 µm), Seaplaque low melting temperature agarose (Lonza), propidium iodide (Beyotime, 50 µg mL^−1^), antibodies against MLF2 for western blot and immunoprecipitation (Santa Cruz, sc‐166881,1:200), MLF2 for immunohistochemistry (Santa Cruz, sc‐166881,1:50), MLF2 for immunofluorescence (Proteintech, 11835‐1‐AP, 1:100; Santa Cruz, sc‐166881, 1:50), USP7 (Bethyl, A300‐033A, 1:1000), glyceraldehyde‐3‐phosphate dehydrogenase (GAPDH) (Santa Cruz, sc‐166545, 1:5000), green fluorescent protein (GFP) (Santa Cruz, sc‐9996, 1:1000), Flag (Proteintech, 20543‐1‐AP, 1:4000), hemagglutinin (HA) (Sigma, H9658, 1:4000), p53 for western blot (Santa Cruz, sc‐126, 1:1000), p53 for immunofluorescence (Santa Cruz, sc‐126, 1:50), p53 for immunohistochemistry (Santa Cruz, sc‐47698, 1:50), Mdm2 (Santa Cruz, sc‐965, 1:500), p21 (Sigma, P1484, 1:5000), ubiquitin (Cell Signaling, #3936, 1:1000), horseradish peroxidase (HRP)‐conjugated secondary antibodies against mouse (115‐035‐062, 1:10 000) and rabbit (111‐035‐144, 1:10 000) (Jackson ImmunoResearch), rhodamine‐conjugated secondary antibody against rabbit (111‐025‐144, 1:200) (Jackson ImmunoResearch), FITC‐conjugated secondary antibody against mouse (115‐095‐146, 1:200) (Jackson ImmunoResearch), normal mouse IgG (Santa Cruz, sc‐2025), and rabbit IgG (Santa Cruz, sc‐2027).

### Cell Culture

HEK293T, HCT116, RKO, SW480, U2OS, NCM460, hTERT–BJ, and Mdm2^−/−^p53^−/−^ MEF cells were maintained in Dulbecco's modified Eagle's medium (DMEM) (Gibco) supplemented with 10% fetal bovine serum (FBS) and 1% penicillin/streptomycin. A549 cells were cultured in Roswell Park Memorial Institute (RPMI) 1640 medium (Gibco) with 10% FBS and 1% penicillin/streptomycin. Mycoplasma contamination of all cell lines was routinely monitored.

### Production and Infection of Lentivirus

The lentiviruses expressing Flag‐tagged MLF2 or the indicated proteins were generated by transfection of pSin‐EF1α‐based constructs together with psPAX2 and pMD2.G into HEK293T cells. To generate lentiviruses expressing the indicated short hairpin RNAs (shRNAs), HEK293T cells were transfected with shRNA (cloned in PLKO.1), pREV, pGag/Pol/PRE, and pVSVG. For generation of the control virus, pSin empty vector and PLKO.1 containing scramble shRNA were used. 12 h after transfection, cells were cultured for an additional 24 h. The lentivirus‐containing culture medium was then collected and used for target cell infection. The shRNA target sequences used in this study are listed in Table S2 (Supporting Information).

### Real‐Time Reverse Transcriptase‐Polymerase Chain Reaction (RT‐PCR)

Real‐time RT‐PCR was performed as previously described.^[^ ^49^ ^]^ The primer sequences are listed in Table S2 (Supporting Information).

### Protein Expression and Purification

The DNA sequence encoding MLF2, p53, or TUBEs (consisting of four tandem repeats of the ubiquitin‐associated (UBA) domains of ubiquilin 1 or human RAD23 homolog A (HR23A)) was individually cloned into the pGEX‐6P‐1 vector. The construct was transformed into *Escherichia coli* BL21 (DE3) cells. After induction with 0.2 mm isopropyl‐d‐1‐thiogalactopyranoside for 20 h at 16 °C, GST‐tagged MLF2, p53, ubiquilin 1 TUBE, or HR23A TUBE proteins were purified by glutathione affinity chromatography.

To purify Flag‐tagged MLF2, p53, and USP7, pRK5‐based plasmids encoding these proteins were transfected into HEK293T cells. Cell lysates were then immunoprecipitated with anti‐Flag M2 beads. To remove nonspecific binding proteins, the beads were sequentially washed with lysis buffer containing 0.25, 0.5, and 1 m KCl as previously described.^[^ ^50^ ^]^ The bound Flag‐tagged proteins were eluted with 3 × Flag peptide.

To purify ubiquitinated GST–p53, the pRK5‐based plasmid encoding GST–p53 was transfected into HEK293T cells. Cells were then treated with MG132 (20 µm) for 6 h to accumulate polyubiquitinated GST–p53, followed by purification using glutathione affinity chromatography.

### Co‐Immunoprecipitation and GST Pull‐Down

For co‐immunoprecipitation (IP) assay, cells were treated with MG132 (20 µm) for 6 h before being lysed in IP buffer (50 mm Tris‐HCl, pH 7.4, 150 mm NaCl, 1.5 mm MgCl_2_, 1 mm EDTA, 0.5% NP‐40, 0.5% Triton X‐100, 10% glycerol, 20 µm MG132, and 1 × protease inhibitor cocktail) by gentle sonication. Cell lysates were precleared with protein A/G agarose beads for 2 h and immunoprecipitated with the indicated antibodies. The input and immunoprecipitates were then analyzed by western blotting.

For GST pull‐down assay, GST–fusion proteins immobilized on glutathione beads were incubated with the indicated proteins in pull‐down buffer (50 mm Tris‐HCl, pH 7.4, 150 mm NaCl, and 0.5% Triton X‐100) for 4 h at 4 °C. Input and bead‐bound proteins were separated by sodium dodecyl sulphate‐polyacrylamide gel electrophoresis (SDS‐PAGE), followed by western blot analysis.

### In Vivo Ubiquitination Assay

Cells were lysed by boiling in denaturing buffer (150 mm Tris‐HCl, pH 8.0, 1% SDS, and 30% glycerol) for 10 min. Cell lysates were then diluted 10 times with buffer A (50 mm Tris‐HCl, pH 8.0, 150 mm NaCl, 0.5% NP‐40, 1× protease inhibitor cocktail, and 2 mm *N*‐ethylmaleimide). After incubation with anti‐p53‐conjugated beads at 4 °C overnight, the immunoprecipitates were analyzed by western blot with antiubiquitin antibody. Alternatively, cells were lysed in denaturing buffer (6 m guanidine–HCl, 0.1 m Na_2_HPO_4_/NaH_2_PO_4_, and 10 mm imidazole, pH 8.0). Cell lysates were incubated with Ni–NTA (nitrilotriacetic acid) agarose beads to pull down proteins conjugated to His‐ubiquitin. Beads‐bound proteins were then analyzed by western blotting with anti‐p53 antibody.

### In Vitro Deubiquitination Assay

Purified ubiquitinated GST–p53 was incubated with Flag–USP7 or Flag–USP7 plus Flag–MLF2 proteins in deubiquitination buffer (50 mm Tris‐HCl, pH 7.6, 50 mm NaCl, 1 mm EDTA, and 10 mm dithiothreitol (DTT)). The reaction mixtures were incubated at 37 °C for 3 h, followed by western blot analysis.

### TUBE Pull‐Down Assay

The TUBE pull‐down assay was performed as previously described.^[^ ^51^ ^]^ Briefly, HCT116 cells were treated with 20 µm MG132 for 6 h before they were lysed in buffer (50 mm Tris‐HCl, pH 7.4, 150 mm NaCl, 1.5 mm MgCl_2_, 1 mm EDTA, 0.5% NP‐40, 0.5% Triton X‐100, 10% glycerol, 2 mm *N*‐ethylmaleimide, 20 µm MG132, and 1 × protease inhibitor cocktail) by gentle sonication. Cell lysates were incubated with GST–TUBEs immobilized on glutathione beads for 4 h at 4 °C. The input and immunoprecipitates were then analyzed by western blotting.

### PLA

Proximity ligation assays were conducted by using Duolink In Situ Red Starter Kit (Sigma) according to the manufacturer's instructions. Briefly, 4% paraformaldehyde‐fixed cells were permeabilized with 0.1% Triton X‐100 for 5 min and then blocked with Duolink blocking solution (Sigma) for 60 min at 37 °C. The cells were incubated with specific primary antibodies at 4 °C overnight, followed by conjugation with PLA oligonucleotides. The isotype‐matched rabbit and mouse IgG were used as negative control. After ligation, amplification, and washing, the cells were stained with DAPI (Vector Laboratories). The PLA signal was acquired using a Zeiss LSM 980 microscope.

### Luciferase Reporter Assay

To determine the effect of MLF2 on the transcriptional activity of p53, HCT116 cells expressing control shRNA or MLF2 shRNA or HCT116 cells expressing control or Flag–MLF2 were transfected with pGL3 control vector, pGL3–p21, or pGL3–Noxa together with Renilla luciferase plasmid. 24 h later, firefly and Renilla luciferase activities were measured by the dual‐luciferase reporter assay system (Promega), and Renilla activity was used to normalize firefly activity.

### Colony Formation in Soft Agar

HCT116 and hTERT–BJ cells were infected with the indicated lentiviruses. 48 h after infection, 5 × 10^3^ cells were suspended in 1.5 mL DMEM containing 10% FBS and 0.3% Seaplaque low melting temperature agarose (Lonza), and plated on top of a 1.5 mL solidified layer of DMEM/10% FBS/0.6% agarose. HCT116 and hTERT–BJ cells were cultured at 37 °C for 2 and 3 weeks, respectively, before they were fixed and stained with crystal violet. The colonies were then scored under a microscope.

### Xenograft Mouse Model

A total of 2 × 10^6^ HCT116 cells transduced with the indicated lentiviruses were subcutaneously injected into the left or right flank of 4 week old BALB/c nude mice (Shanghai SLAC Laboratory Animal Co. Ltd.) (*n* = 6 per group). After injection, tumor volumes were measured every 6 days with a caliper and calculated using the equation: volume = length × width^2^ × 0.52. 24 days after injection, the mice were sacrificed and tumors were excised and weighed. The extracted proteins from the excised tumors were analyzed by western blotting.

### Immunohistochemistry

Colorectal adenocarcinoma (CRC) and normal tissues were acquired from surgical colectomy specimens, which were collected from 88 patients with stage I–III CRC between August and December 2015 at the First Affiliated Hospital of Anhui Medical University (Hefei, Anhui Province, China). Clinical data were recorded and updated retrospectively. Another set of 33 colorectal adenocarcinoma specimens carrying wild‐type *TP53* was obtained from CRC patients who underwent colorectal resection at the First Affiliated Hospital of Anhui Medical University between August 2020 and October 2022.

For immunohistochemistry assay, the isolated human colorectal adenocarcinoma tissue and matched adjacent normal tissue were immersed in formalin overnight and then embedded in paraffin. Paraffin‐embedded tissues were sectioned and the tissue slides were deparaffinized with xylene and rehydrated through an ethanol gradient. Then, antigen retrieval and inactivation of endogenous peroxidase were performed before samples were incubated with the indicated primary antibodies at 4 °C overnight. The slides were then reacted with secondary antibody and stained with diaminobenzidine substrate. The tissue slides were visualized and captured using an upright microscope (Nikon Eclipse Ni). The immunohistochemical staining intensity scores were indicated as: negative (0), weak staining (1), moderate staining (2), and strong staining (3), and the extent of stained cells were indicated as: 0% = 0, 1–25% = 1, 26–50% = 2, 51–75% = 3, 76–100% = 4. The final scores ranging from 0–12 were defined by multiplying the intensity scores by the scores of the extent of stained cells. Based on the final scores, the immunoreactivity was classified as: negative, 0–3; weak positive, 4–6; strong positive, 7–9; and extrastrong positive, 10–12.

### Ethics Statement

All experiments with human tissue specimens were approved by the Ethics Committee of the First Affiliated Hospital of Anhui Medical University (PJ 2023‐07‐48), and all participants provided written informed consent. The animal experiments were approved by the Animal Research Ethics Committee of the University of Science and Technology of China (USTCACUC22120122091). All studies were performed in accordance with relevant guidelines and regulations.

### Reproducibility

All experiments were repeated at least 3 times with similar results. The shown images were representative of three independent experiments.

### Statistical Analysis

Statistical analysis was carried out using Microsoft Excel software and GraphPad Prism to assess differences between experimental groups. Statistical significance was analyzed by two‐tailed Student's *t*‐test or one‐way analysis of variance (ANOVA) and expressed as a *p* value. *p* < 0.05 was considered to be statistically significant. *, **, *** indicated *p* < 0.05, *p* < 0.01, *p* < 0.001, respectively; ns. indicated no significance.

## Conflict of Interest

The authors declare no conflict of interest.

## Author Contributions

D.F., H.H., and K.Z. contributed equally to this work. D.F., H.H., K.Z., and Y.M. designed research. D.F., H.H., K.Z., N.Y., B.Y., S.T. performed research. A.X., C.Y., and Y.Z. provided clinical samples. D.F., H.H., K.Z., A.X., C.Y., Y.Z., N.Y., B.Y., S.T., X.W., and Y.M. analyzed data. D.F., K.Z. and Y.M. wrote the paper.

## Supporting information

Supporting Information

## Data Availability

The data that support the findings of this study are available from the corresponding author upon reasonable request.
