# Supplementary material for: MLF2 Negatively Regulates P53 and Promotes Colorectal Carcinogenesis
Source: Adv Sci (Weinh). 2023 Jul 12;10(26):2303336. doi: 10.1002/advs.202303336 (PMC10502657; doi:10.1002/advs.202303336)
Supplement: Supplementary file 1 — Supporting Information [file ADVS-10-2303336-s001.pdf]

ADVANCED SCIENCE
Open Access

Supporting Information

for *Adv. Sci.*, DOI 10.1002/advs.202303336

MLF2 Negatively Regulates P53 and Promotes Colorectal Carcinogenesis

Debao Fang, Hao Hu, Kailiang Zhao, Aman Xu, Changjun Yu, Yong Zhu, Ning Yu, Bo Yao, Suyun Tang, Xianning Wu and Yide Mei**

Supplementary Figure Legends

Figure S1. Related to Figure 1.

(A) HCT116 cells expressing Flag-p53 and HA-MLF2 were subjected to proximity ligation assay (PLA) using anti-Flag and anti-HA antibodies. The isotype-matched rabbit (R) and mouse (M) IgG were also used as negative control. The PLA signal was acquired using a Zeiss LSM 980 microscope (Top). Numbers of puncta were also counted (n = 100 cells) (Down).

(B) Schematic representation of wild-type MLF2 and its deletion mutants used for mapping experiments.

(C) HEK293T cells were transfected with Flag-p53 together with the indicated GFP-MLF2 constructs. Cell lysates were subjected to immunoprecipitation with anti-Flag antibody, followed by western blot analysis.

(D) Schematic representation of wild-type p53 and its deletion mutants used for mapping experiments.

(E) HEK293T cells were transfected with HA-MLF2 alone or together with the indicated Flag-p53 constructs. Cell lysates were subjected to immunoprecipitation with anti-Flag antibody, followed by western blot analysis.

Figure S2. Related to Figure 2.

(A and B) SW480 cells were infected with lentiviruses expressing control shRNA, MLF2 shRNA#1, or MLF2 shRNA#2 (A). SW480 cells were infected with lentiviruses expressing control or Flag-MLF2 (B). Forty-eight hours later, cell lysates were analyzed by western blotting.

(C and D) HCT116 cells were infected with lentiviruses expressing control shRNA or MLF2 shRNA (C). HCT116 cells were infected with lentiviruses expressing control or Flag-MLF2 (D). Twenty-four hours later, cells were transfected with the indicated luciferase reporter constructs. Reporter activity was then measured 24 h after transfection. ***, $p < 0.001$; ns., no significance.

(E and F) HCT116 cells were infected with lentiviruses expressing control shRNA or

MLF2 shRNA (E). HCT116 cells were infected with lentiviruses expressing control or Flag-MLF2 (F). Forty-eight hours later, total RNA was analyzed by real-time RT-PCR. Data shown are mean ± SD (n=3). *, $p < 0.05$; **, $p < 0.01$; ***, $p < 0.001$; ns., no significance.

(G) The blots in Figure 2C were quantified by Image J software and the ratio of p53 to GAPDH was then calculated. The data are mean ± SD from three independent experiments.

(H) The blots in Figure 2D were quantified by Image J software and the ratio of p53 to GAPDH was then calculated. The data are mean ± SD from three independent experiments.

(I and J) HCT116 cells with knockdown (I) or overexpression (J) of MLF2 were treated with MG132 (20 μM) for 6 h. Cell lysates were then incubated with GST-TUBEs (HR23A) immobilized on glutathione beads. Input and bead-bound proteins were analyzed by western blotting.

Figure S3. Related to Figure 3.

(A) HCT116 cells expressing Flag-USP7 and HA-MLF2 were subjected to proximity ligation assay (PLA) using anti-Flag and anti-HA antibodies. The isotype-matched rabbit (R) and mouse (M) IgG were also used as negative control. The PLA signal was acquired using a Zeiss LSM 980 microscope (Top). Numbers of puncta were also counted (n = 100 cells) (Down).

(B) Immunofluorescence staining of HEK293T cells transfected with HA-MLF2 (green), Flag-USP7 (red), or both HA-MLF2 (green) and Flag-USP7 (red). The images were taken with a fluorescence microscope (Leica DMI600 B).

(C) Schematic representation of wild-type MLF2 and its deletion mutants. HEK293T cells were transfected with Flag-USP7 together with the indicated GFP-MLF2 constructs. Cell lysates were subjected to immunoprecipitation with anti-Flag antibody, followed by western blot analysis.

(D and E) Schematic representation of wild-type USP7 and its deletion mutants.

HEK293T cells were transfected with GFP-MLF2 alone or together with the indicated Flag-USP7 constructs. Cell lysates were subjected to immunoprecipitation with anti-Flag antibody, followed by western blot analysis.

Figure S4. Related to Figure 5.

(A and B) The growth curves of RKO cells expressing control or Flag-MLF2 (A). The expression of MLF2 was detected by western blot analysis (B). Data shown are mean ± SD (n=3). **, $p < 0.01$.

(C and D) The growth curves of RKO cells expressing control shRNA, MLF2 shRNA, p53 shRNA, or MLF2 shRNA plus p53 shRNA (C). The knockdown efficiency of MLF2 and p53 was verified by western blot analysis (D). Data shown are mean ± SD (n=3). ***, $p < 0.001$.

(E) The representative images for Figure 5E.

(F and G) (F) RKO cells expressing control or Flag-MLF2 were treated with doxorubicin (Dox; 0.5 μg/ml) for the indicated periods of time. Cells were co-stained with Annexin V-FITC and Hoechst 33342, and Annexin V-positive cells were counted as apoptotic cells. Data shown are mean ± SD (n=3). Cell lysates were analyzed by western blotting with the indicated antibodies. CL-PARP and CL-Cas-3 indicate cleaved PARP and cleaved caspase-3, respectively. ***, $p < 0.001$. (G) The representative images were also shown.

(H) The representative images for Figure 5F.

(I and J) (I) RKO cells expressing control shRNA, MLF2 shRNA, p53 shRNA, or MLF2 shRNA plus p53 shRNA were treated with doxorubicin (Dox; 0.5 μg/ml) for the indicated periods of time. Cells were co-stained with Annexin V-FITC and Hoechst 33342, and Annexin V-positive cells were counted as apoptotic cells. Cell lysates were analyzed by western blotting with the indicated antibodies. Data shown are mean ± SD (n=3). CL-PARP and CL-Cas-3 indicate cleaved PARP and cleaved caspase-3, respectively. ***, $p < 0.001$. (J) The representative images were also shown.

(K-M) (K) The growth curves of SW480 cells expressing control or Flag-MLF2. (L)

SW480 cells expressing control or Flag-MLF2 were assayed for their ability to form colonies in soft agar. Images were taken 2 weeks after cell seeding. Numbers of colonies in six randomly selected areas (40 × magnification) were counted and averaged. (M) The expression of MLF2 was detected by western blot analysis. Data shown are mean ± SD (n=3). **, $p < 0.01$; ***, $p < 0.001$.

(N-P) (N) The growth curves of SW480 cells expressing control shRNA, MLF2 shRNA#1, or MLF2 shRNA#2. (O) SW480 cells expressing control shRNA, MLF2 shRNA#1, or MLF2 shRNA#2 were assayed for their ability to form colonies in soft agar. Images were taken 2 weeks after cell seeding. Numbers of colonies in six randomly selected areas (40 × magnification) were counted and averaged. (P) The knockdown efficiency of MLF2 was verified by western blot analysis. Data shown are mean ± SD (n=3). ***, $p < 0.001$.

Figure S5. Related to Figure 6.

(A-D) A total of 2×10^6 HCT116 cells transduced with lentiviruses expressing control or Flag-MLF2 were individually injected into nude mice (n = 6 for each group). Xenograft tumors were taken four 24 days after injection (A). Excised tumors were weighed (B). Tumor sizes were measured at the indicated time points (C). Protein extracts from the excised xenografts were also analyzed by western blotting (D). **, $p < 0.01$; ***, $p < 0.001$.

Figure S1

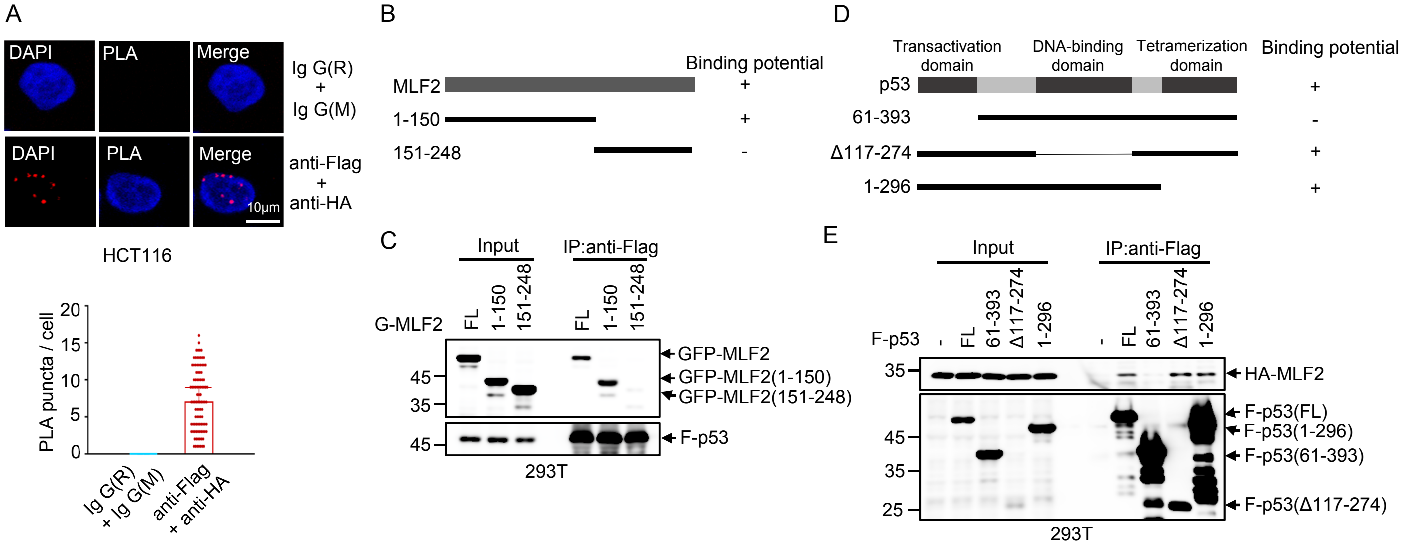
A
DAPI
PLA
Merge
Ig G(R)
+
Ig G(M)
anti-Flag
+
anti-HA
10μm
HCT116
PLA puncta / cell
0
5
10
15
20
Ig G(R) + Ig G(M)
anti-Flag + anti-HA
B
Binding potential
MLF2
1-150
151-248
+
+
-
C
Input
IP:anti-Flag
G-MLF2
FL
1-150
151-248
45
35
GFP-MLF2
GFP-MLF2(1-150)
GFP-MLF2(151-248)
F-p53
293T
D
Transactivation domain
DNA-binding domain
Tetramerization domain
Binding potential
p53
61-393
Δ117-274
1-296
+
-
+
+
E
Input
IP:anti-Flag
F-p53
-
FL
61-393
Δ117-274
1-296
35
45
25
HA-MLF2
F-p53(FL)
F-p53(1-296)
F-p53(61-393)
F-p53(Δ117-274)
293T

Figure S2

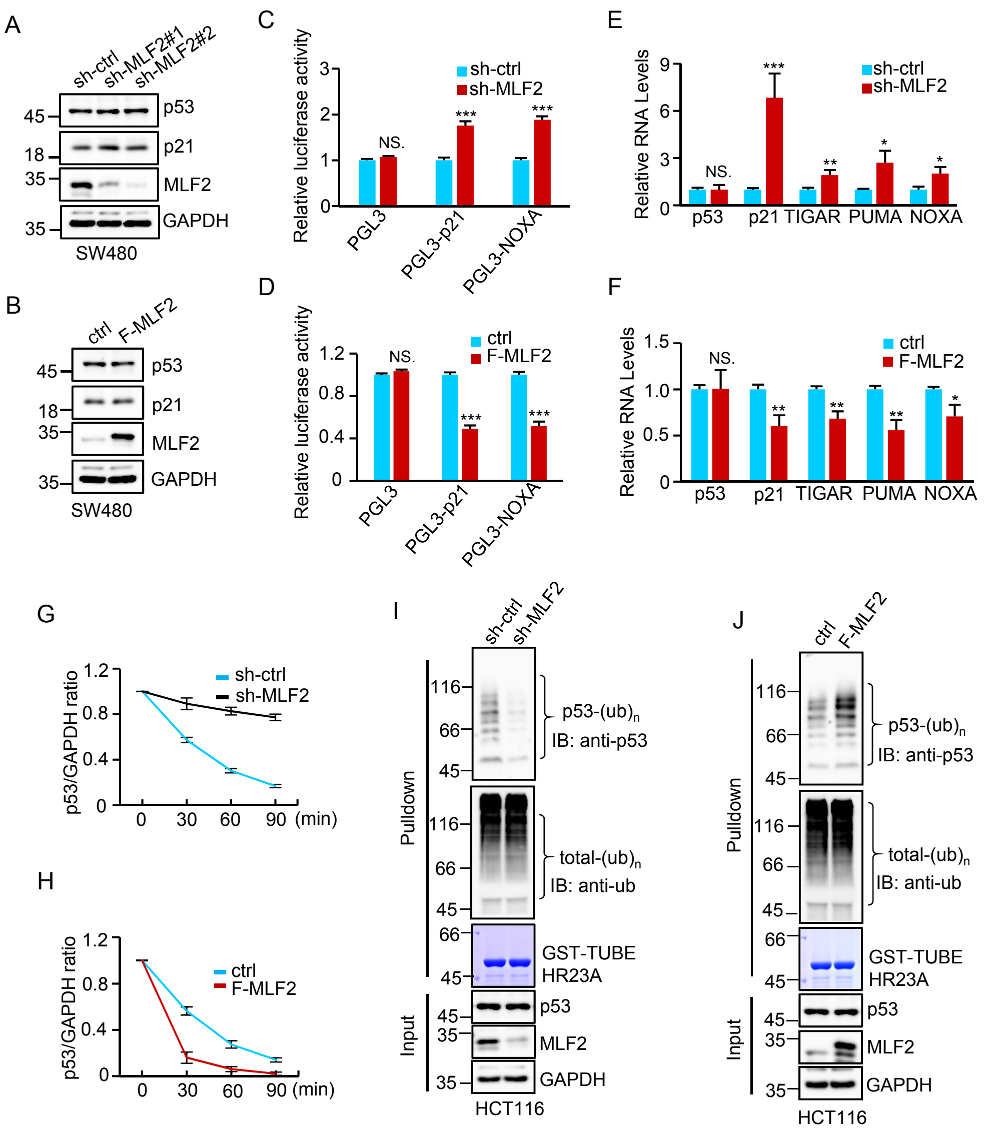

A
sh-ctrl
sh-MLF2#1
sh-MLF2#2
p53
p21
MLF2
GAPDH
SW480
B
ctrl
F-MLF2
SW480
C
Relative luciferase activity
sh-ctrl
sh-MLF2
NS.
PGL3
PGL3-p21
PGL3-NOXA
D
ctrl
F-MLF2
E
Relative RNA Levels
p53
p21
TIGAR
PUMA
NOXA
F
G
p53/GAPDH ratio
(min)
H
I
Pulldown
Input
p53-(ub)n
IB: anti-p53
total-(ub)n
IB: anti-ub
GST-TUBE
HR23A
HCT116
J

Figure S3

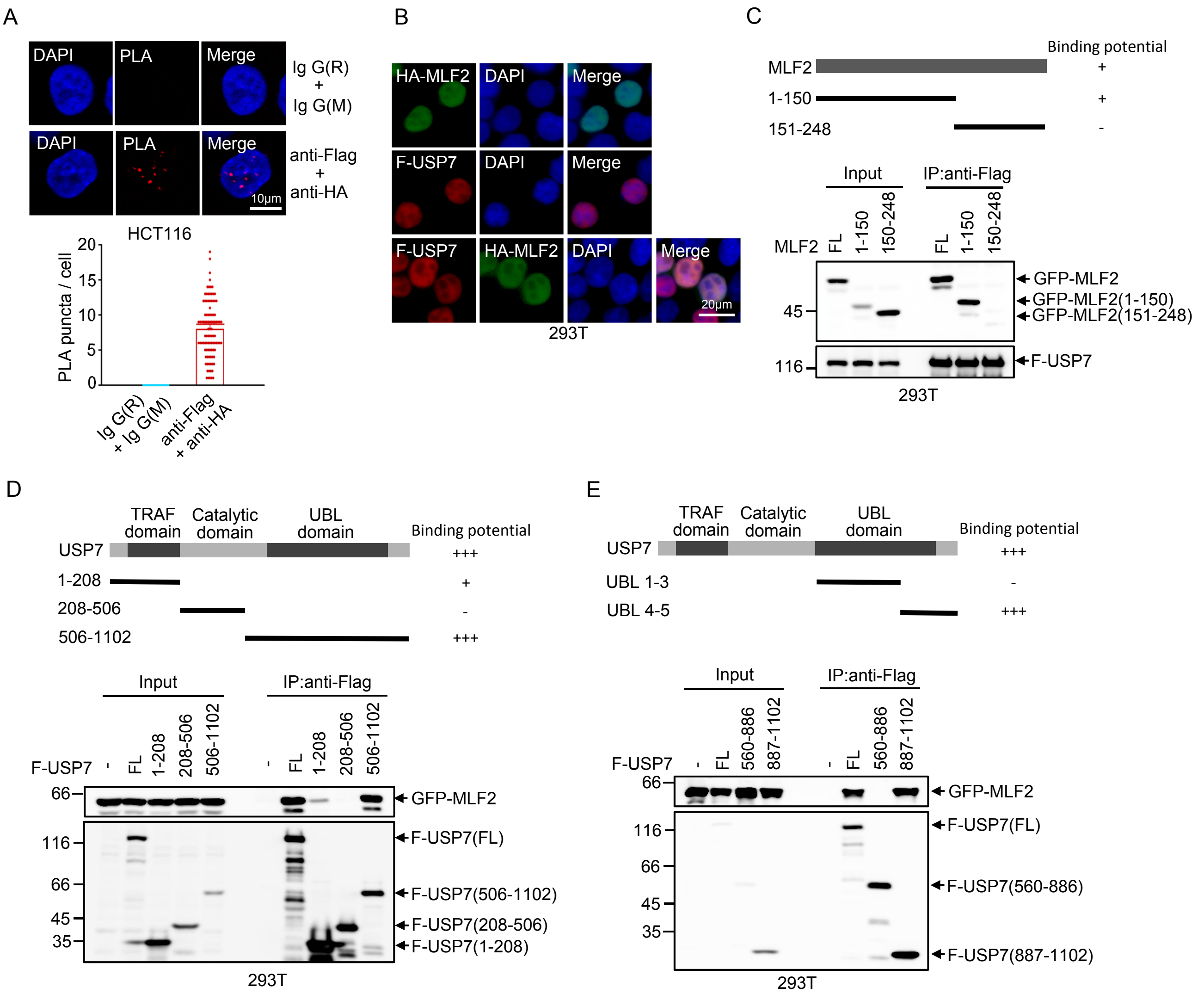
A
DAPI
PLA
Merge
Ig G(R)
+
Ig G(M)
anti-Flag
+
anti-HA
10μm
HCT116
PLA puncta / cell
Ig G(R) + Ig G(M)
anti-Flag + anti-HA
B
HA-MLF2
DAPI
Merge
F-USP7
20μm
293T
C
Binding potential
MLF2
1-150
151-248
Input
IP:anti-Flag
FL
150-248
GFP-MLF2
GFP-MLF2(1-150)
GFP-MLF2(151-248)
F-USP7
293T
D
TRAF domain
Catalytic domain
UBL domain
USP7
1-208
208-506
506-1102
+++
F-USP7(FL)
F-USP7(506-1102)
F-USP7(208-506)
F-USP7(1-208)
E
UBL 1-3
UBL 4-5
560-886
887-1102
F-USP7(560-886)
F-USP7(887-1102)

Figure S4

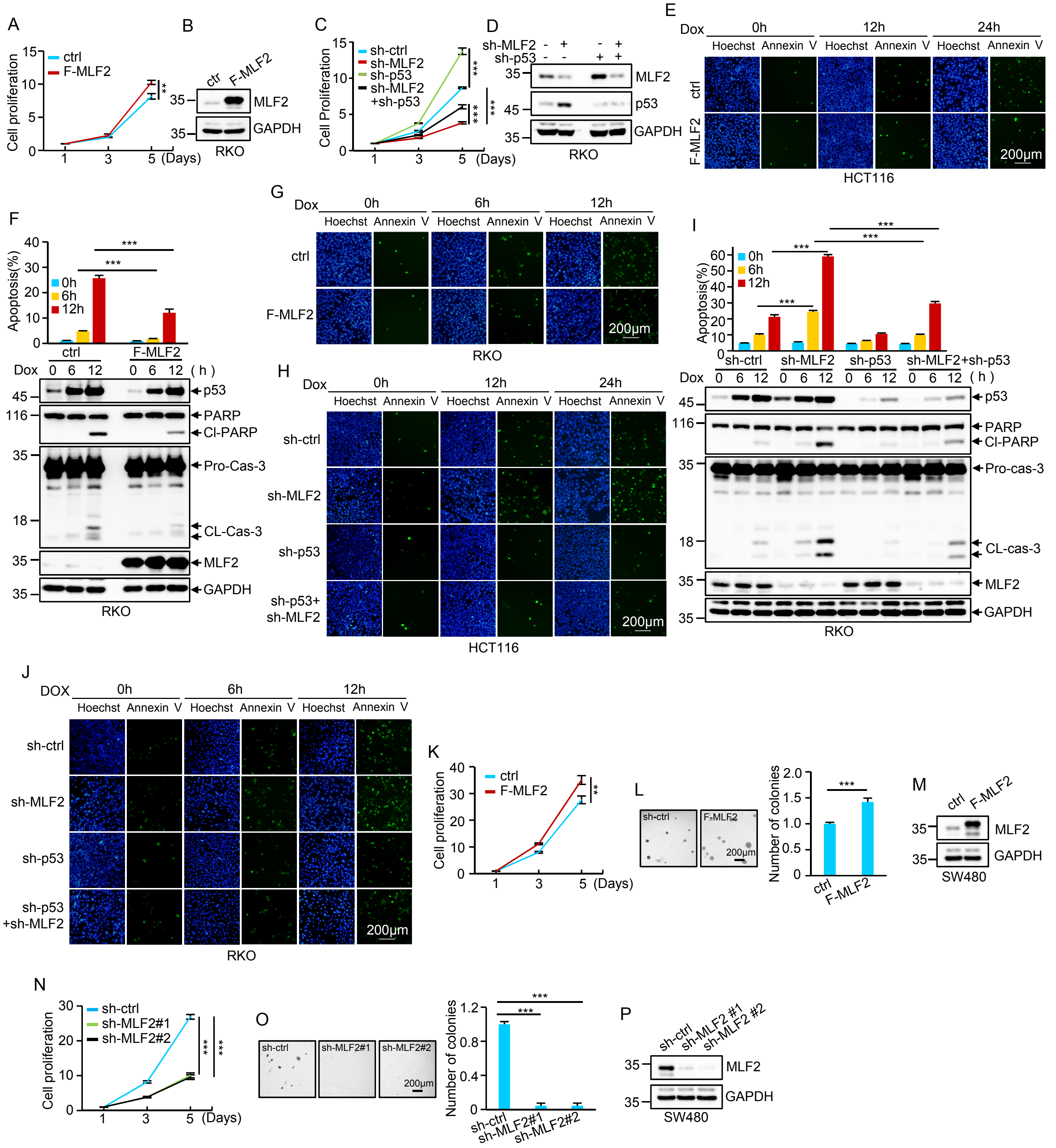

A
Cell proliferation
ctrl
F-MLF2
(Days)
B
ctr
F-MLF2
MLF2
GAPDH
RKO
C
Cell Proliferation
sh-ctrl
sh-MLF2
sh-p53
sh-MLF2 +sh-p53
(Days)
D
sh-MLF2
sh-p53
MLF2
p53
GAPDH
RKO
E
Dox
0h
12h
24h
Hoechst
Annexin V
ctrl
F-MLF2
200μm
HCT116
F
Apoptosis(%)
0h
6h
12h
ctrl
F-MLF2
Dox
p53
PARP
Cl-PARP
Pro-Cas-3
CL-Cas-3
MLF2
GAPDH
RKO
G
Dox
0h
6h
12h
Hoechst
Annexin V
ctrl
F-MLF2
200μm
RKO
H
Dox
0h
12h
24h
Hoechst
Annexin V
sh-ctrl
sh-MLF2
sh-p53
sh-p53+ sh-MLF2
200μm
HCT116
I
Apoptosis(%)
0h
6h
12h
sh-ctrl
sh-MLF2
sh-p53
sh-MLF2+sh-p53
Dox
p53
PARP
Cl-PARP
Pro-cas-3
CL-cas-3
MLF2
GAPDH
RKO
J
DOX
0h
6h
12h
Hoechst
Annexin V
sh-ctrl
sh-MLF2
sh-p53
sh-p53 +sh-MLF2
200μm
RKO
K
Cell proliferation
ctrl
F-MLF2
(Days)
L
sh-ctrl
F-MLF2
200μm
Number of colonies
ctrl
F-MLF2
M
ctrl
F-MLF2
MLF2
GAPDH
SW480
N
Cell proliferation
sh-ctrl
sh-MLF2#1
sh-MLF2#2
(Days)
O
sh-ctrl
sh-MLF2#1
sh-MLF2#2
200μm
Number of colonies
sh-ctrl
sh-MLF2#1
sh-MLF2#2
P
sh-ctrl
sh-MLF2 #1
sh-MLF2 #2
MLF2
GAPDH
SW480

Figure S5

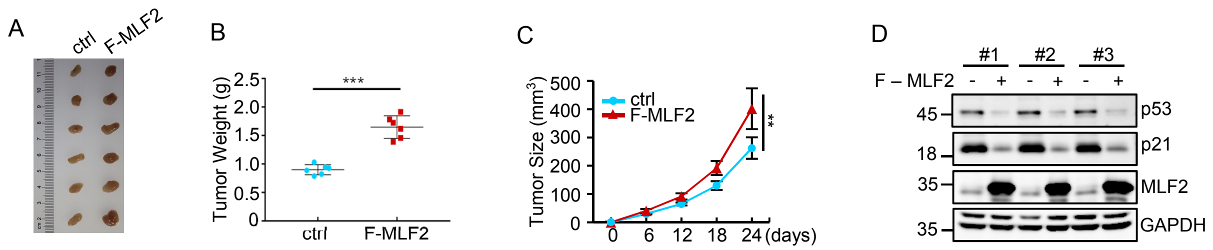
A
ctrl
F-MLF2
B
Tumor Weight (g)
2.5
2.0
1.5
1.0
0.5
0
***
ctrl
F-MLF2
C
Tumor Size (mm^3)
500
400
300
200
100
0
ctrl
F-MLF2
**
0
6
12
18
24
(days)
D
#1
#2
#3
F – MLF2
- + - + - +
45
p53
18
p21
35
MLF2
35
GAPDH

Table S1: Proteins in anti-p53 immunoprecipitates identified by mass spectrometry

Gene name	Reference	PepCount	UniquePepCount	CoverPercent	MW	PI
TP53	sp|P04637|P53_HUMAN Cellular tumor antigen p53 OS=Homo sapiens OX=9606 PE=2 SV=1	15	13	36.13%	43753.9	6.48
HNRNPK	sp|P61978|HNRPK_HUMAN Heterogeneous nuclear ribonucleoprotein K OS=Homo sapiens OX=9606 GN=HNRNPK PE=1 SV=1	21	14	37.37%	50975.7	5.39
DDX5	sp|P17844|DDX5_HUMAN Probable ATP-dependent RNA helicase DDX5 OS=Homo sapiens OX=9606 GN=DDX5 PE=1 SV=1	16	15	24.10%	69147.3	9.06
ANXA1	sp|P04083|ANXA1_HUMAN Annexin A1 OS=Homo sapiens OX=9606 GN=ANXA1 PE=1 SV=2	19	12	43.35%	38713.8	6.57
RANBP2	sp|P49792|RBP2_HUMAN E3 SUMO-protein ligase RanBP2 OS=Homo sapiens OX=9606 GN=RANBP2 PE=1 SV=2	13	12	4.62%	358195	5.86
SSRP1	sp|Q08945|SSRP1_HUMAN FACT complex subunit SSRP1 OS=Homo sapiens OX=9606 GN=SSRP1 PE=1 SV=1	13	12	16.50%	81073.9	6.45
KHSRP	sp|Q92945|FUBP2_HUMAN Far upstream element-binding protein 2 OS=Homo sapiens OX=9606 GN=KHSRP PE=1 SV=4	13	12	20.53%	73114.5	6.85
HUWE1	sp|Q7Z6Z7|HUWE1_HUMAN E3 ubiquitin-protein ligase HUWE1 OS=Homo sapiens OX=9606 GN=HUWE1 PE=1 SV=3	12	12	3.54%	481885	5.1
RPS3	sp|P23396|RS3_HUMAN 40S ribosomal protein S3 OS=Homo sapiens OX=9606 GN=RPS3 PE=1 SV=2	23	11	47.33%	26688	9.68
RPL7A	sp|P62424|RL7A_HUMAN 60S ribosomal protein L7a OS=Homo sapiens OX=9606 GN=RPL7A PE=1 SV=2	15	11	32.33%	29995.2	10.61
MARS	sp|P56192|SYMC_HUMAN Methionine--tRNA ligase, cytoplasmic OS=Homo sapiens OX=9606 GN=MARS PE=1 SV=2	12	11	13.11%	101115	5.82
MIB1	sp|Q86YT6|MIB1_HUMAN E3 ubiquitin-protein ligase MIB1 OS=Homo sapiens OX=9606 GN=MIB1 PE=1 SV=1	12	11	12.82%	110135	6.47
UHRF1	sp|Q96T88|UHRF1_HUMAN E3 ubiquitin-protein ligase UHRF1 OS=Homo sapiens OX=9606 GN=UHRF1 PE=1 SV=1	12	11	15.01%	89812.9	7.66
HYOU1	sp|Q9Y4L1|HYOU1_HUMAN Hypoxia up-regulated protein 1 OS=Homo sapiens OX=9606 GN=HYOU1 PE=1 SV=1	12	11	12.31%	111334	5.16
GANAB	sp|Q14697|GANAB_HUMAN Neutral alpha-glucosidase AB OS=Homo sapiens OX=9606 GN=GANAB PE=1 SV=3	11	11	13.14%	106873	5.74
LDHA	sp|P00338|LDHA_HUMAN L-lactate dehydrogenase A chain OS=Homo sapiens OX=9606 GN=LDHA PE=1 SV=2	17	10	28.92%	36688.3	8.44
MCM7	sp|P33993|MCM7_HUMAN DNA replication licensing factor MCM7 OS=Homo sapiens OX=9606 GN=MCM7 PE=1 SV=4	12	10	14.60%	81307.1	6.08
TRIM28	sp|Q13263|TIF1B_HUMAN Transcription intermediary factor 1-beta OS=Homo sapiens OX=9606 GN=TRIM28 PE=1 SV=5	12	10	13.17%	88548.8	5.52
RUVBL2	sp|Q9Y230|RUVB2_HUMAN RuvB-like 2 OS=Homo sapiens OX=9606 GN=RUVBL2 PE=1 SV=3	12	10	23.11%	51156	5.49
HNRNPC	sp|P07910|HNRPC_HUMAN Heterogeneous nuclear ribonucleoproteins C1/C2 OS=Homo sapiens OX=9606 GN=HNRNPC PE=1 SV=4	12	10	24.51%	33669.6	4.95
PSMD1	sp|Q99460|PSMD1_HUMAN 26S proteasome non-ATPase regulatory subunit 1 OS=Homo sapiens OX=9606 GN=PSMD1 PE=1 SV=2	11	10	13.33%	105835	5.25
MATR3	sp|P43243|MATR3_HUMAN Matrin-3 OS=Homo sapiens OX=9606 GN=MATR3 PE=1 SV=2	10	10	12.63%	94622.2	5.87
SERBP1	sp|Q8NC51|PAIRB_HUMAN Plasminogen activator inhibitor 1 RNA-binding protein OS=Homo sapiens OX=9606 GN=SERBP1 PE=1 SV=2	10	10	23.77%	44964.8	8.66
GCN1	sp|Q92616|GCN1_HUMAN eIF-2-alpha kinase activator GCN1 OS=Homo sapiens OX=9606 GN=GCN1 PE=1 SV=6	10	10	3.78%	292755	7.29
AHCY	sp|P23526|SAHH_HUMAN Adenosylhomocysteinase OS=Homo sapiens OX=9606 GN=AHCY PE=1 SV=4	11	9	18.75%	47715.6	5.92
NUMA1	sp|Q14980|NUMA1_HUMAN Nuclear mitotic apparatus protein 1 OS=Homo sapiens OX=9606 GN=NUMA1 PE=1 SV=2	10	9	4.35%	238257	5.63
CDK1	sp|P06493|CDK1_HUMAN Cyclin-dependent kinase 1 OS=Homo sapiens OX=9606 GN=CDK1 PE=1 SV=3	9	9	34.01%	34095	8.38
DDB1	sp|Q16531|DDB1_HUMAN DNA damage-binding protein 1 OS=Homo sapiens OX=9606 GN=DDB1 PE=1 SV=1	9	9	7.37%	126966	5.14
CPNE3	sp|O75131|CPNE3_HUMAN Copine-3 OS=Homo sapiens OX=9606 GN=CPNE3 PE=1 SV=1	8	8	13.41%	60130	5.6
FUBP1	sp|Q96AE4|FUBP1_HUMAN Far upstream element-binding protein 1 OS=Homo sapiens OX=9606 GN=FUBP1 PE=1 SV=3	8	8	13.98%	67559.6	7.18
BAG6	sp|P46379|BAG6_HUMAN Large proline-rich protein BAG6 OS=Homo sapiens OX=9606 GN=BAG6 PE=1 SV=2	8	7	6.71%	119407	5.4
NUP93	sp|Q8N1F7|NUP93_HUMAN Nuclear pore complex protein Nup93 OS=Homo sapiens OX=9606 GN=NUP93 PE=1 SV=2	7	7	9.04%	93487.2	5.5
LRRC59	sp|Q96AG4|LRC59_HUMAN Leucine-rich repeat-containing protein 59 OS=Homo sapiens OX=9606 GN=LRRC59 PE=1 SV=1	7	6	18.89%	34930.1	9.61
TRIM27	sp|P14373|TRI27_HUMAN Zinc finger protein RFP OS=Homo sapiens OX=9606 GN=TRIM27 PE=1 SV=1	7	6	15.40%	58489.1	5.83
NQO1	sp|P15559|NQO1_HUMAN NAD(P)H dehydrogenase [quinone] 1 OS=Homo sapiens OX=9606 GN=NQO1 PE=1 SV=1	8	5	20.07%	30867.3	8.91
PRMT5	sp|O14744|ANM5_HUMAN Protein arginine N-methyltransferase 5 OS=Homo sapiens OX=9606 GN=PRMT5 PE=1 SV=4	6	5	6.28%	72683	5.88
HDAC1	sp|Q13547|HDAC1_HUMAN Histone deacetylase 1 OS=Homo sapiens OX=9606 GN=HDAC1 PE=1 SV=1	4	4	8.30%	55102.4	5.31
G3BP2	sp|Q9UN86|G3BP2_HUMAN Ras GTPase-activating protein-binding protein 2 OS=Homo sapiens OX=9606 GN=G3BP2 PE=1 SV=2	4	4	9.96%	54120.5	5.41
TXNL1	sp|O43396|TXNL1_HUMAN Thioredoxin-like protein 1 OS=Homo sapiens OX=9606 GN=TXNL1 PE=1 SV=3	4	4	25.26%	32251	4.84
MLF2	sp|Q15773|MLF2_HUMAN Myeloid leukemia factor 2 OS=Homo sapiens OX=9606 GN=MLF2 PE=1 SV=1	3	3	10.48%	28147.1	6.4
USP7	sp|Q93009|UBP7_HUMAN Ubiquitin carboxyl-terminal hydrolase 7 OS=Homo sapiens OX=9606 GN=USP7 PE=1 SV=2	3	3	2.45%	128301	5.33
CDK2	sp|P24941|CDK2_HUMAN Cyclin-dependent kinase 2 OS=Homo sapiens OX=9606 GN=CDK2 PE=1 SV=2	3	3	11.07%	33929.1	8.8
UBE3A	sp|Q05086|UBE3A_HUMAN Ubiquitin-protein ligase E3A OS=Homo sapiens OX=9606 GN=UBE3A PE=1 SV=4	3	3	3.77%	100686	5.12
STUB1	sp|Q9UNE7|CHIP_HUMAN E3 ubiquitin-protein ligase CHIP OS=Homo sapiens OX=9606 GN=STUB1 PE=1 SV=2	2	2	8.91%	34855.8	5.61
AURKA	sp|O14965|AURKA_HUMAN Aurora kinase A OS=Homo sapiens OX=9606 GN=AURKA PE=1 SV=2	2	2	4.47%	45808.8	9.45
FBXO46	sp|Q6PJ61|FBX46_HUMAN F-box only protein 46 OS=Homo sapiens OX=9606 GN=FBXO46 PE=1 SV=3	2	2	2.32%	64630.1	7.19
MDM2	sp|Q00987|MDM2_HUMAN E3 ubiquitin-protein ligase Mdm2 OS=Homo sapiens OX=9606 GN=MDM2 PE=1 SV=1	1	1	3.26%	55232.3	4.6
USP21	sp|Q9UK80|UBP21_HUMAN Ubiquitin carboxyl-terminal hydrolase 21 (Fragment) OS=Homo sapiens OX=9606 GN=USP21 PE=4 SV=1	1	1	13.51%	4287.9	6.28

Table S2. Oligonucleotides used in this study

**Primers used in qRT-PCR assays**	
p53	FW: 5' GCCATCTACAAGCAGTCACAG 3'
	REV: 5' TCATCCAAATACTCCACACGC 3'
p21	FW: 5' TGTCACTGTCTTGTACCCTTG 3'
	REV: 5' GGCGTTTGGAGTGGTAGAA 3'
TIGAR	FW: 5' GGAAGAGTGCCCTGTGTTTAC 3'
	REV: 5' AGTTGCTTGGAGATCCTTGG 3'
PUMA	FW: 5' CGACCTCAACGCACAGTAC 3'
	REV: 5' CCTAATTGGGCTCCATCTCG 3'
NOXA	FW: 5' GGAGATGCCTGGGAAGAAG 3'
	REV: 5' CCTAATTGGGCTCCATCTCG 3'
GAPDH	FW: 5' CCATGGGGAAGGTGAAGGTC 3'
	REV: 5' TGCCGGAAGTTCAGTTTGTC 3'
**Oligonucleotide sequence of shRNAs**	
sh-MLF2#1	5' CCTTCTTTAATCTCCTGGTTT 3'
sh-MLF2#2	5' GTGGTTTCATGGACATGTTTG 3'
sh-p53	5' GACTCCAGTGGTAATCTAC 3'
sh-USP7	5' CCTGGATTTGTGGTTACGTTA 3'
sh-control	5' CCTAAGGTTAAGTCGCCCTCG 3'
